# Robotic and laparoscopic liver surgery for colorectal liver metastases: an experience from a German Academic Center

**DOI:** 10.1186/s12957-020-02113-1

**Published:** 2020-12-22

**Authors:** Mirhasan Rahimli, Aristotelis Perrakis, Vera Schellerer, Andrew Gumbs, Eric Lorenz, Mareike Franz, Jörg Arend, Victor-Radu Negrini, Roland Siegfried Croner

**Affiliations:** 1 Department of General, Visceral, Vascular and Transplant Surgery, University Hospital Magdeburg, Leipziger Str. 44, 39120 Magdeburg, Germany; 2 Department of Pediatric Surgery, University Hospital Erlangen, Krankenhausstraße 12, 91054 Erlangen, Germany; 3 Department of Surgery, Centre Hospitalier Intercommunal de Poissy/Saint-Germain-en-Laye, 10 Rue du Champ Gaillard, 78300 Poissy, France

**Keywords:** Robotic surgery, Laparoscopic surgery, Liver resection, Surgical oncology, Colorectal liver metastases, Da Vinci

## Abstract

**Background:**

Minimally invasive liver surgery (MILS) in the treatment of colorectal liver metastases (CRLM) is increasing in incidence. The aim of this work was to present our experience by reporting short-term and long-term outcomes after MILS for CRLM with comparative analysis of laparoscopic (LLS) and robotic liver surgery (RLS).

**Methods:**

Twenty-five patients with CRLM, who underwent MILS between May 2012 and March 2020, were selected from our retrospective registry of minimally invasive liver surgery (MD-MILS). Thirteen of these patients underwent LLS and 12 RLS. Short-term and long-term outcomes of both groups were analyzed.

**Results:**

Operating time was significantly longer in the RLS vs. the LLS group (342.0 vs. 200.0 min; *p* = 0.004). There was no significant difference between the laparoscopic vs. the robotic group regarding length of postoperative stay (8.8 days), measured blood loss (430.4 ml), intraoperative blood transfusion, overall morbidity (20.0%), and liver surgery related morbidity (4%). The mean BMI was 27.3 (range from 19.2 to 44.8) kg/m^2^. The 30-day mortality was 0%. R0 resection was achieved in all patients (100.0%) in RLS vs. 10 patients (76.9%) in LLS. Major resections were carried out in 32.0% of the cases, and 84.0% of the patients showed intra-abdominal adhesions due to previous abdominal surgery. In 24.0% of cases, the tumor was bilobar, the maximum number of tumors removed was 9, and the largest tumor was 8.5 cm in diameter. The 1-, 3- and 5-year overall survival rates were 84, 56.9, and 48.7%, respectively. The 1- and 3-year overall recurrence-free survival rates were 49.6 and 36.2%, respectively, without significant differences between RLS vs. LLS.

**Conclusion:**

Minimally invasive liver surgery for CRLM is safe and feasible. Minimally invasive resection of multiple lesions and large tumors is also possible. RLS may help to achieve higher rates of R0 resections. High BMI, previous abdominal surgery, and bilobar tumors are not a barrier for MILS. Laparoscopic and robotic liver resections for CRLM provide similar long-term results which are comparable to open techniques.

## Background

Liver metastases can be diagnosed in about 40% of patients with colorectal cancer. Approximately 20% of patients are diagnosed with synchronous liver metastases at the time of the initial diagnosis [1]. However, this is not a contraindication to surgery, because modern surgery combined with chemotherapy can achieve a 5-year survival rate up to 60% [2].

The use of minimally invasive liver surgery (MILS) is increasing worldwide. But currently there is still not enough evidence to determine its value in the treatment of colorectal liver metastases (CRLM). Open liver surgery is still estimated as a standard procedure in the surgical therapy of CRLM. However, CRLM are increasingly being removed via minimally invasive approaches because of the potential benefit to patients [2–4].

A meta-analysis showed that laparoscopic CRLM resection was associated with significantly higher R0 resection rates, less intra-operative blood loss, fewer blood transfusions, less overall morbidity, and shorter length of hospitalization, but with longer operative times when compared to open surgery. There was no significant difference between laparoscopic and open groups regarding the long-term outcomes [2]. In a recent multicenter study, comparable perioperative and long-term oncologic outcomes of robotic and laparoscopic surgery for colorectal liver metastases were identified [4]. Furthermore, another multicenter study figured out a 3-year disease-free and overall survival of 41.9 and 66.1% in patients who underwent robotic liver surgery for CRLM, respectively. The overall and major morbidity in this study were 27 and 5%, respectively [5]. These data highlight the value of both minimally invasive techniques, i.e., robotics and laparoscopy in surgery for CRLM.

Even the simultaneous minimally invasive resection of colorectal liver metastases and the primary tumor can be performed safely and effectively [6–8]. A recent meta-analysis showed that simultaneous minimally invasive resection of colorectal cancer and synchronous liver metastases caused significantly lower intra-operative blood loss and blood transfusion, faster recovery of intestinal function and diet, shorter length of stay, and lower rates of surgical complications in comparison to open surgery. In this study, the two procedures did not differ significantly in terms of other perioperative results, disease-free survival, or overall survival [1].

The aim of our study was to present the short-term and long-term outcomes of our patients after minimally invasive surgery for CRLM with a comparative analysis of laparoscopic and robotic liver resections.

## Methods

### Patients

From the Magdeburg registry of minimally invasive liver surgery (MD-MILS), patients were selected who underwent minor and major minimally invasive liver surgery for CRLM between May 2012 and March 2020. Other types of liver metastases were not considered for this study. Hybrid and hand port-assisted techniques were excluded from the study. We made no selection regarding intrahepatic tumor localization, number of lesions, tumor size, body mass index (BMI), or previous abdominal surgery. The cases requiring vascular reconstruction or multivisceral resection were not selected for minimally invasive liver surgery.

We identified 25 patients in our registry regarding the selection criteria. The patient cohort was divided in two groups. The first group consisted of 13 patients, who underwent laparoscopic liver surgery (LLS), and the second group consisted of 12 patients, who underwent robotic liver surgery (RLS).

### Definitions

The resection of ≥ 3 segments was defined as a major resection, while the resection of one or two liver segments was considered a minor resection. Overall morbidity was defined as all intraoperative and postoperative complications. Liver surgery related complications included posthepatectomy liver failure, intraoperative and postoperative bleeding, bile leak, bile fistula, bilioma, cholangitis, cholangiosepsis, liver abscess, and portal vein thrombosis. The severity of the complications were assessed according to the Clavien-Dindo classification [9]. As length of stay (LOS), we defined the duration of the postoperative hospital stay. We defined the patient**’**s death within 30 postoperative days as a 30-day mortality. Final diagnosis and number of intrahepatic lesions were evaluated based on histopathological examination.

### Minimally invasive liver surgery

The liver parenchymal dissection during laparoscopic surgery was performed using the water jet or the ultrasonic dissectors. We used the Da Vinci Si System and since September 2019 the Da Vinci Xi system (Intuitive Surgical, Inc., Sunnyvale, CA, USA) for robotic liver surgery. The dissection of liver parenchyma was carried out with the harmonic scalpel, in combination with the bipolar forceps or with monopolar scissors in combination with the Waterjet (ERBEJET® 2, Erbe Elektromedizin GmbH, Tübingen, Germany) during robotic liver surgery.

### Statistical analysis

We analyzed patient characteristics, perioperative parameters, type of procedures, and tumor characteristics in the entire cohort and between the two groups (LLS vs. RLS).

In addition, we investigated perioperative outcomes of major and minor resections in the entire cohort and as a subgroup analysis in the robotic approach. We also compared minor laparoscopic and robotic liver resections as a subgroup analysis. Furthermore, we analyzed the long-term oncological outcomes after minimally invasive resection of CRLM.

The patient data were collected retrospectively. Data analysis was performed using the IBM SPSS Statistics for Windows, Version 26 (IBM Corp., Armonk, NY, USA). Cross tables were used for the descriptive analysis of categorical variables. We used the number of cases and percentages for data presentation. We applied Fisher’s exact test for the significance test for dichotomous variables. The independent samples *t* test or Mann-Whitney *U* test were applied for the continuous variables. The data was presented using the mean and standard deviation (SD).

For survival analysis, we used the Kaplan-Meier method to determine overall and recurrence-free survival. The log-rank test was applied to evaluate for any significant differences between the laparoscopic and robotic groups. We used the median survival in months with standard error (SE) and 95% confidence interval (CI) to present the survival data. One-, 3-, and 5-year survival rates were given as percentages.

Statistical significance was considered at a *p* value of < 0.05.

## Results

### Patient characteristics and perioperative outcomes

The patient characteristics and perioperative outcomes of our cohort is summarized in Table 1. Sixteen male (64.0%) and nine female (36.0%) patients formed our cohort. While the distribution of the sexes was equal in the robotic group, in the laparoscopic group the men predominated with 10 patients (76.9%). The patients were 62.8 (SD 11.8) years old on average. The patients in our study were mainly overweight. The mean body mass index (BMI) was 27.3 (SD 5.8) kg/m^2^. There was no significant difference between the laparoscopic and the robotic group regarding the sex, age, and BMI. Twenty-one patients (84.0%) in our study had previously undergone abdominal surgery and were found at the liver resection to have resultant intra-abdominal adhesions. Ten (76.9%) of them were in the laparoscopic group and 11 (91.7%) patients in the robotic one. There was no significant difference between the two groups.

**Table 1:** Patient demographics and perioperative outcomes in patients with colorectal liver metastasis who underwent minor and major minimally invasive liver surgery (MILS)

	LLS *n* (% or SD)	RLS *n* (% or SD)	*p* value	Total *n* (% or SD)
Total	13 (52.0)	12 (48.0)		25 (100.0)
Sex				
Male	10 (76.9)	6 (50.0)	0.226	16 (64.0)
Female	3 (23.1)	6 (50.0)		9 (36.0)
Age (years)	62.1 (12.6)	63.5 (11.3)	0.770	62.8 (11.8)
BMI (kg/m^2^)	28.3 (7.6)	26.2 (2.7)	0.894	27.3 (5.8)
Operating time^a^ (min)	200.0 (116.8)	342.0 (101.4)	**0.004**	268.2 (129.5)
LOS (days)	8.5 (3.4)	9.3 (4.2)	0.852	8.8 (3.7)
MBL (ml)	412.3 (529.1)	450.0 (278.0)	0.225	430.4 (419.3)
Intraoperative blood transfusion	3 (23.1)	2 (16.7)	1.000	5 (20.0)
Overall morbidity	2 (15.4)	3 (25.0)	0.645	5 (20.0)
Liver surgery related morbidity	0 (0.0)	1 (8.3)	0.480	1 (4.0)
Previous abdominal surgery	10 (76.9)	11 (91.7)	0.593	21 (84.0)
R status				
R0	10 (76.9)	12 (100.0)	0.220	22 (88.0)
R1	3 (23.1)	0 (0.0)		3 (12.0)
Major resections	3 (23.1)	5 (41.7)	0.411	8 (32.0)
Minor resections	10 (76.9)	7 (58.3)		17 (68.0)

*BMI* body mass index, *MBL* measured blood loss, *LLS* laparoscopic liver surgery, *LOS* length of postoperative stay, *MILS* minimally invasive liver surgery, *RLS* robotic liver surgery, *SD* standard deviation

^a^The operating time in the robotic group is excluding the docking time

The mean operating time was 200.0 (SD 116.8) and 342.0 (101.4) min in the laparoscopic vs. the robotic group, which was significantly different (*p* = 0.004). The mean overall length of postoperative stay and measured blood loss was 8.8 (SD 3.7) days and 430.4 (SD 419.3) ml, respectively, without significant difference between the laparoscopic and the robotic groups. Five patients (20.0%) received intraoperative blood transfusions. Three (23.1%) of them were in the laparoscopic group and two (16.7%) in the robotic one without a significant difference between the two groups.

We detected five overall complications (20.0%). Two complications (15.4%) were in the laparoscopic group. Both of these patients developed a wound infection (Clavien-Dindo grade I). The remaining three complications (25.0%) were in the robotic group. One patient developed an enterocutaneous fistula from the small intestine postoperatively which did not need surgery (Clavien-Dindo grade II). The second patient developed a lymphocele, which also did not need operative intervention (Clavien-Dindo grade II). The third complication in the robotic group was a postoperative bile leak after right hemihepatectomy (Clavien-Dindo grade IIIa). This complication was the only liver-specific surgery related complication (8.3%, overall 4.0%) in our study. There was no significant difference in overall and liver surgery related morbidity between the laparoscopic and robotic groups.

### Type of surgical procedures

We performed eight major (32.0%) and 17 (68.0%) minor minimally invasive liver resections. We recorded three major (23.1%) and 10 (76.9%) minor resections in the laparoscopic group. The proportion of major resections was higher in the robotic group with five patients (41.7%). Seven robotic minor resections (58.3%) were performed in this group. There was no significant difference between the robotic and laparoscopic groups regarding the distribution of minor and major resections (Table 1).

As shown in the Table 2, we performed three left (12.0%) and two right (8.0%) hemi-hepatectomies in our study. One patient (4.0%) underwent the anatomical resection of four liver segments and atypical resection of the caudate lobe. The remaining two patients (8.0%), who underwent major liver resections, underwent resection of three liver segments. Furthermore, we carried out eight left lateral liver resections (32.0%) and six anatomical segmentectomies (24.0%). Other minor resections were two bisegmentectomies (8.0%) and one anatomical resection of two non-contiguous liver segments (4.0%).

**Table 2:** Surgical procedures in patients with colorectal liver metastasis who underwent minor and major minimally invasive liver surgery (MILS)

	LLS *n* (%)	RLS *n* (%)	Total *n* (%)
Major resections			
Left hemi-hepatectomy	0 (0.0)	3 (25.0)	3 (12.0)
Right hemi-hepatectomy	1 (7.7)	1 (8.3)	2 (8.0)
Resection of 3 segments	1 (7.7)	1 (8.3)	2 (8.0)
Resection > 3 segments	1 (7.7)	0 (0.0)	1 (4.0)
Minor resections			
Left lateral LR	4 (30.8)	4 (33.3)	8 (32.0)
Anatomical resection 1 segment	4 (30.8)	2 (16.7)	6 (24.0)
Bisegmentectomy	1 (7.7)	1 (8.3)	2 (8.0)
Anatomical 2 segment resection	1 (7.7)	0 (0.0)	1 (4.0)
Total	13 (100.0)	12 (100.0)	25 (100.0)

*LLS* laparoscopic liver surgery, *LR* liver resection, *MILS* minimally invasive liver surgery, *RLS* robotic liver surgery

### Tumor characteristics

In 22 patients (88.0%) of our study, we achieved a R0 resection. Three patients (12.0%) showed a microscopically positive resection margin. All of these patients were in the laparoscopic group. In the robotic group, rate of R0 resections was 100.0%. But the difference between the two groups was not statistically significant (*p* = 0.220) (Table 1). Twenty patients (80.0%) had already been treated with chemotherapy at the time of the operation. The mean value of perioperative carcinoembryonic antigen (CEA) was 112.4 (303.1) ng/ml and was higher in the robotic group than in the laparoscopic one. However, this difference was not statistically significant.

As shown in the Table 3, most lesions were located in the left lobe of the liver. We recorded 12 cases (48.0%) with the tumor localized in the left hepatic lobe. Seven patients (28.0%) presented with disease in the right hepatic lobe. In six cases (24.0%), the tumor localization was bilobar. The number of liver lesions was confirmed on histopathology. Fourteen patients (56.0%) showed a single lesion, six patients (24.0%) 2 lesions, two patients (8.0%) 3 lesions, and three patients (12.0%) had more than 3 lesions. The highest number of liver lesions was 9. Hepatic metastases presented synchronously in 10 cases (40.0%) and metachronously in 13 cases (52.0%). The mean longest diameter of the largest lesion was 2.8 (SD 1.9) mm and 4.2 (SD 1.6) mm in the laparoscopic and robotic groups, respectively. The largest tumor was 8.5 cm in longest diameter.

**Table 3:** Tumor characteristics in patients with colorectal liver metastasis who underwent minor and major minimally invasive liver surgery (MILS)

	LLS *n* (% or SD)	RLS *n* (% or SD)	Total *n* (% or SD)
Tumor localization			
Left hepatic lobe	6 (46.2)	6 (50.0)	12 (48.0)
Right hepatic lobe	3 (23.1)	4 (33.3)	7 (28.0)
Bilobar	4 (30.8)	2 (16.7)	6 (24.0)
Number of lesions			
1	8 (61.5)	6 (50.0)	14 (56.0)
2	3 (23.1)	3 (25.0)	6 (24.0)
3	1 (7.7)	1 (8.3)	2 (8.0)
> 3	1 (7.7)	2 (16.7)	3 (12.0)
Time of occurrence			
Synchronous	6 (46.2)	4 (33.3)	10 (40.0)
Metachronous	5 (38.5)	8 (66.7)	13 (52.0)
Missing data	2 (15.4)	0 (0.0)	2 (8.0)
Size of tumor (cm)	2.8 (1.9)	4.2 (1.6)	3.5 (1.8)
Preoperative chemotherapy			
Yes	10 (76.9)	10 (83.3)	20 (80.0)
No	3 (23.1)	1 (8.3)	4 (16.0)
Missing data	0 (0.0)	1 (8.3)	1 (4.0)
CEA (ng/ml)	40.1 (51.8)	171.5 (404.8)	112.4 (303.1)

*LLS* laparoscopic liver surgery, *MILS* minimally invasive liver surgery, *CEA* carcinoembryonic antigen, *RLS* robotic liver surgery, *SD* standard deviation

### Comparison of minor and major minimally invasive liver resections

In addition, we performed a comparative analysis of the perioperative outcomes of major and minor resections. Table 4 shows the comparison of preoperative outcomes among minor and major minimally invasive liver resections. The mean operating time was 223.2 (103.8) min in the minor and 363.6 (132.6) min in the major MILS group, respectively. This difference was statistically significant (*p* = 0.008). On average, the patients spent 7.6 (SD 3.0) days in the hospital after minor resections and 11.5 (SD 3.9) days after major resections, respectively. This difference was statistically significant (*p* = 0.007). The minor group had significantly (*p* = 0.013) lower mean measured blood loss (274.1 ml) than the major group (762.5 ml). In terms of overall and liver surgery related morbidity and R-status, there was no significant difference between the two groups.

**Table 4:** Comparison of minor and major minimally invasive liver resections regarding perioperative outcomes in patients with colorectal liver metastasis

	Minor MILS *n* (% or SD)	Major MILS *n* (% or SD)	*p* value
Total	17 (68.0)	8 (32.0)	
Operating time^a^ (min)	223.2 (103.8)	363.6 (132.6)	**0.008**
LOS (days)	7.6 (3.0)	11.5 (3.9)	**0.007**
MBL (ml)	274.1 (259.5)	762.5 (513.2)	**0.013**
Overall morbidity	3 (17.6)	2 (25.0)	1.000
Liver surgery related morbidity	0 (0.0)	1 (12.5)	0.320
R status			
R0	16 (94.1)	6 (75.0)	0.231
R1	1 (5.9)	2 (25.0)	

*MBL* measured blood loss, *LOS* length of postoperative stay, *MILS* minimally invasive liver surgery, *SD* standard deviation

^a^The operating time in the robotic group is excluding the docking time

### Comparison of minor and major robotic liver resections

Furthermore, we compared the perioperative outcomes of minor and major robotic liver resections as a subgroup analysis (Table 5). The robotic minor group showed significantly shorter mean operating times (*p* = 0.035) and LOS (*p* = 0.010) than the robotic major group. There was no significant difference between these subgroups regarding the measured blood loss and overall and liver surgery related morbidity.

**Table 5:** Comparison of minor and major robotic liver resections regarding perioperative outcomes in patients with colorectal liver metastasis as a subgroup analysis

	Minor RLS *n* (% or SD)	Major RLS *n* (% or SD)	*p* value
Total	7	5	
Operating time^a^ (min)	292.0 (83.5)	412.0 (85.6)	**0.035**
LOS (days)	7.0 (2.2)	12.4 (4.5)	**0.010**
MBL (ml)	342.9 (280.5)	600.0 (215.1)	0.117
Overall morbidity	1 (14.3)	2 (40.0)	0.523
Liver surgery related morbidity	0 (0.0)	1 (20.0)	0.417
R status			
R0	7 (100.0)	5 (100.0)	–
R1	0 (0.0)	0 (0.0)	

*MBL* measured blood loss, *LOS* length of postoperative stay, *RLS* robotic liver surgery, *SD* standard deviation

^a^The operating time in the robotic group is excluding the docking time

### Comparison of laparoscopic vs. robotic minor liver resections

Moreover, we performed a comparative analysis of perioperative outcomes after laparoscopic and robotic minor liver resections (Table 6). Only the operating time was significantly shorter (*p* = 0.017) in the laparoscopic minor group than in the robotic minor group. In terms of the LOS, measured blood loss, morbidity, and R status, we could not detect any significant difference between these subgroups.

**Table 6:** Comparison of laparoscopic and robotic minor liver resections regarding perioperative outcomes in patients with colorectal liver metastasis as a subgroup analysis

	Minor LLS *n* (% or SD)	Minor RLS *n* (% or SD)	*p* value
Total	10	7	
Operating time^a^ (min)	175.1 (90.8)	292.0 (83.5)	**0.017**
LOS (days)	8.0 (3.6)	7.0 (2.2)	0.669
MBL (ml)	226.0 (247.0)	342.9 (280.5)	0.270
Overall morbidity	2 (20.0)	1 (14.3)	1.000
Liver surgery related morbidity	0 (0.0)	0 (0.0)	–
*R* status			
R0	9 (90.0)	7 (100.0)	1.000
R1	1 (10.0)	0 (0.0)	

*MBL* measured blood loss, *LLS* laparoscopic liver surgery, *LOS* length of postoperative stay, *RLS* robotic liver surgery, *SD* standard deviation

^a^The operating time in the robotic group is excluding the docking time

### Overall and recurrence-free survival

Finally, we investigated the long-term outcomes of our patients after minimally invasive liver surgery due to the CRLM. The median overall survival was 47 months (SE 22.2, 95% CI 3.5–90.5). The 1-, 3-, and 5-year overall survival was 84, 56.9, and 48.7%, respectively. The median overall survival in the laparoscopic group was 47 months (SE 18.7, 95% CI 10.4–83.6) and in the robotic groups 29 months (SE 9.0, 95% CI 11.4–46.6). There was no significant difference between the groups regarding the overall survival (*p* = 0.733). The 1- and 3-year overall survival was 70% vs. 100% and 60% vs. 44.4% in the laparoscopic vs. the robotic group, respectively.

The median overall recurrence-free survival (RFS) was 11 months (SE 7.7, 95% CI 0–26.2). The 1- and 3-year overall RFS were 49.6% and 36.2%, respectively. The median overall RFS in the laparoscopic cohort was 24 months (SE 12.2, 95 % CI 0.2–47.8) and in the robotic group 11 months (SE 8.9, 95% CI 0–28.5), respectively. The groups did not differ significantly in terms of RFS (*p* = 0.646). The 1- and 3-year overall RFS were 54.9% vs. 44.4% and 41.1% vs. 33.3% in the laparoscopic vs. the robotic group, respectively.

## Discussion

The utilization of minimally invasive surgery for CRLM is increasing worldwide. The first randomized controlled trial comparing laparoscopic and open liver resection for CRLM was performed by Fretland et al. This study, including 147 open vs. 133 laparoscopic patients, showed that laparoscopic resections were associated with a significantly lower postoperative complication rate and shorter LOS. In terms of blood loss, operating time, resection margins, and 90-day postoperative mortality, there was no significant difference between the open and laparoscopic groups [10]. A recent multicenter study comparing robotic and laparoscopic surgery for colorectal liver metastases demonstrated largely comparable short-term and long-term outcomes after laparoscopic and robotic resections [4].

Our cohort consisted of non-selected cases regarding the patient conditions and tumor characteristics. Only the operating time was significantly longer (*p* = 0.004) in the robotic group when compared to the laparoscopic group in our study. The overall mean operating time was 268.2 (129.5). This finding was comparable with other studies [4, 11]. There was no significant difference in our study between the laparoscopic and robotic groups regarding the remaining short-term outcomes which is consistent with other studies. Nevertheless, there was a trend towards higher R0 resections in the robotic group.

Our patients showed a mean BMI value of 27.3 kg/m^2^ (range = 19.2 to 44.8 kg/m^2^). The highest BMI was 44.8 kg/m^2^ in our cohort. Here, we can only corroborate the previous findings that high BMI is not a contraindication for minimally invasive liver surgery [12, 13]. The LOS in our study was longer (8.5 days in the laparoscopic and 9.3 days in the robotic group) than the abovementioned studies [4, 10, 11]. One reason for this is that the German DRG (Diagnosis Related Groups) system needs a LOS between 5 and 19 days to get sufficient reimbursement for patient care after liver surgery. The blood loss in our cohort was higher than in other studies [10, 14]. This can be explained by the high rate of previous abdominal surgery causing adhesions (84.0%) and major resections (32.0%) in our study. Furthermore, we never used a Pringle maneuver during liver surgery in this study.

Overall and liver surgery related morbidity were 20.0 and 4.0% in our study, respectively. Our complication rates were comparable with the literature. Other studies report an overall complication rate of 19 to 29.6% after MILS [4, 5, 10, 11, 15, 16]. The mortality rate for MILS is reported in the literature to range from 0 to 0.3% [4, 5, 15, 16]. No patient died in our series. This is an indicator of the safety of minimally invasive liver surgery. R positive resections were detected in 7% of cases in open cases and in 6% in the laparoscopic group in the abovementioned randomized controlled study [10]. The multi-center study published by Beard et al. demonstrated R1 resection rates of 16.7 and 20.0% in the robotic and laparoscopic groups, respectively. R2 rates of 3.5 and 0.9% were also shown in the robotic and laparoscopic groups, respectively [4]. Overall in three patients (12.0%), we detected a microscopic positive resection margin (R1). All of these patients were in the laparoscopic group. In the robotic group, R0 resections were achieved in all cases (100.0%). R2 resections were not noted in our study. Here, we can argue that more precise control of parenchymal dissection during robotic surgery may help to achieve R0 resections more safely.

As can be seen in the other studies and in our work, tumor localization plays no role in the implementation of minimally invasive liver surgery [4, 10]. Both unilateral (right or left hepatic lobe) and bilobar liver lesions can be approached minimally invasively. Twenty percent of the patients in our study showed multiple liver metastases ranging from 3 to 9 lesions. The mean largest tumor size in our study was 3.5 cm (SD 1.8) with the maximum diameter measuring 8.5 cm. We can thus show that minimally invasive resection of multiple lesions or large tumors can be carried out safely and successfully. In Beard’s multicenter study, the tumor size was ≥ 5 cm in 14.2% of robotic cases [4].

The comparison of minor and major minimally invasive liver surgery in our work showed that the outcomes regarding the operating time, LOS, and MBL were significantly better in the minor resection group than in the major resection group. It is quite understandable because of the difference in the extent of liver resection. But there was no significant difference between minor and major groups in terms of overall and liver surgery related complications and R status, which demonstrates the feasibility and safety of the MILS for major liver resections. Moreover, the comparison of minor and major robotic liver resections as subgroup analysis did not show any significant differences between robotic minor and major resection groups regarding the MBL and morbidity. Because R0 resection was achieved in all robotic patients, there was no difference between these subgroups. Here, we can also say that the implementation of robotics for major liver resections is safe and feasible. Admittedly, the small patient cohort and the retrospective nature are the limitations of our study.

Our overall and recurrence-free survival rates are comparable with previous findings [3–5]. As reported in the multicenter study published by Beard et al., we also did not find any significant difference between laparoscopic and robotic groups regarding long-term outcomes [4]. The meta-analyses showed comparable long-term oncological outcomes after minimally invasive and open resection of CRLM [1, 2].

## Conclusion

Minimally invasive liver surgery for CRLM is safe and feasible. Robotic liver surgery may help to achieve higher rates of R0 resections. High BMI, previous abdominal surgery, and bilobar tumor localization are not a barrier for minimally invasive liver surgery. Laparoscopic and robotic liver resections for CRLM provide similar long-term results which are comparable to open techniques.

## Data Availability

The datasets used and/or analyzed during the current study are available from the corresponding author on reasonable request.

## Abbreviations

BMI: Body mass index
CEA: Carcinoembryonic antigen
CI: Confidence interval
MBL: Measured blood loss
LLS: Laparoscopic liver surgery
LOS: Length of postoperative stay
LR: Liver resection
MILS: Minimally invasive liver surgery
RFS: Recurrence free survival
RLS: Robotic liver surgery
SD: Standard deviation
SE: Standard error
